# A Prospective Concept on the Fabrication of Blend PES/PEG/DMF/NMP Mixed Matrix Membranes with Functionalised Carbon Nanotubes for CO_2_/N_2_ Separation

**DOI:** 10.3390/membranes11070519

**Published:** 2021-07-10

**Authors:** Ashvin Viknesh Mahenthiran, Zeinab Abbas Jawad

**Affiliations:** 1 Department of Chemical Engineering, Faculty of Engineering and Science, Curtin University Malaysia, CDT 250, 98009 Miri, Sarawak, Malaysia; 700015933@student.curtin.edu.my; 2 Department of Chemical Engineering, College of Engineering, Qatar University, P.O. Box 2713, Doha, Qatar

**Keywords:** CO_2_/N_2_ separation, blend mixed matrix membrane, polyether sulfone, polyethylene glycol, methyl-2-pyrrolidone, dimethylformamide

## Abstract

With an ever-increasing global population, the combustion of fossil fuels has risen immensely to meet the demand for electricity, resulting in significant increase in carbon dioxide (CO_2_) emissions. In recent years, CO_2_ separation technology, such as membrane technology, has become highly desirable. Fabricated mixed matrix membranes (MMMs) have the most desirable gas separation performances, as these membranes have the ability to overcome the trade-off limitations. In this paper, blended MMMs are reviewed along with two polymers, namely polyether sulfone (PES) and polyethylene glycol (PEG). Both polymers can efficiently separate CO_2_ because of their chemical properties. In addition, blended N-methyl-2-pyrrolidone (NMP) and dimethylformamide (DMF) solvents were also reviewed to understand the impact of blended MMMs’ morphology on separation of CO_2_. However, the fabricated MMMs had challenges, such as filler agglomeration and void formation. To combat this, functionalised multi-walled carbon nanotube (MWCNTs-F) fillers were utilised to aid gas separation performance and polymer compatibility issues. Additionally, a summary of the different fabrication techniques was identified to further optimise the fabrication methodology. Thus, a blended MMM fabricated using PES, PEG, NMP, DMF and MWCNTs-F is believed to improve CO_2_/nitrogen separation.

## 1. Global Warming

An environmental crisis associated with climate change is due to CO_2_ emissions [1]. As a consequence, sea levels are rising, biodiversity is compromised and the animal population is slowly becoming endangered [2,3]. These results are due to anthropogenic sources, which contribute up to 80% of the greenhouse gases (GHGs) [4]. This steady contribution from anthropogenic sources is because of the global population growth [5]. Thus, this results in higher demand for energy and, with an abundant availability of fossil fuels, emissions of CO_2_ will continue to increase as well [5]. According to the Intergovernmental Panel on Climate Change, it has been predicted that by 2035, the CO_2_ levels will rise to 450 ppm, thus causing an increase in global temperature of 2 °C [6].

## 2. Carbon Capture Technologies

Currently, there is a need to implement and retrofit carbon capture systems to new and existing plant designs that assist to capture 90% of emissions and limit the energy costs by 35% [7,8]. As a consequence, many large emission sources have implemented one of the three carbon capture systems (oxy-fuel capture systems, pre-combustion capture systems and post-carbon capture systems) targeting capture performance and minimal energy requirements [9,10].

Oxy-fuel combustion uses a flue gas primarily of CO_2_ and H_2_O, which is created by using pure oxygen rather than ambient air [11]. After undergoing a desulphurisation process, the remaining CO_2_ is captured and stored. Furthermore, the NO_x_ emissions are lower because they are carried out in a nitrogen-depleted environment. As such, this process allows the CO_2_ concentration to be higher at the output stream, thereby creating easier separation of exhaust gases due to the high flame temperature [12]. The main advantage of this system is that it can be installed in power plants, as it has a similar assembly design [13]. However, this system incurs higher capital costs due to the requirements of an oxygen-rich environment [11].

In a pre-combustion system, a conversion process occurs where CO_2_ is the undesired product generated and captured [14]. Both carbon monoxide and hydrogen gases react with oxygen/air in the shift converter, generating an excess of CO_2_ [15]. This system can be divided into chemical and physical absorption processes [16]. A chemical absorption process incurs a higher capital cost due to solvent regeneration, similar to a physical absorption process which uses pressure as its driving mechanism [17]. Therefore, greater partial pressure and a high composition of CO_2_ are observed, which allow better absorption efficiency [18].

A post-combustion capture system has the ability to reduce the concentration of CO_2_ by 5–15% when it is captured from the waste gas stream after conversion from the carbon source [19]. Additionally, higher CO_2_ pressure and are is achieved during capture, requiring more energy and cost [20]. This system utilises four different techniques, namely adsorption, absorption, cryogenic distillation and membranes [21]. By using these techniques, there can be flexibility in operation, as they can be retrofitted into existing plants and, therefore, revisions in the combustion cycle are not required [11]. As such, intensive research and funding have been invested in this promising system [22]. Thus, this system allows plants to reduce sustainable costs as well as the environmental impact of CO_2_ emissions [23]. However, a drawback of this system is the condition of allowing flue gas to enter the CO_2_ concentration in the feed stream [23].

The three systems are potentially helpful in mitigating the global CO_2_ emissions. Nonetheless, post-combustion capture is more advantageous than the other two systems because it can be retrofitted into existing plants, hence lowering the initial capital costs of this mitigation source [22].

## 3. Post-Combustion Capture Technologies

Currently, the post-combustion carbon capture technology is widely applied, as it is the most promising and can be retrofitted into existing power plants [24]. However, capturing the CO_2_ from the flue gas creates a challenge, as the latter consists of other gas components [25]. Further, the effectiveness of post-combustion capture is dependent on CO_2_’s selectivity among the other gas particles. The lower the CO_2_ selectivity, the higher the cost of using this system [26]. Moreover, post-combustion capture has various methodologies to help in the selectivity of CO_2_ through physical and chemical processes, such as absorption, adsorption, cryogenic distillation and membrane separation technologies [27].

### 3.1. Absorption

Absorption is commonly used in industries that have acid-like gases in their stream and, being a mature technology, uses chemical solvents to capture CO_2_ [28]. It is divided into two categories, which are the physical process and the chemical process [11]. The physical process is dependent on pressure and temperature, where the absorption of CO_2_ occurs at high pressure and low temperature [29,30]. Acid–base neutralisation reactions on basic solvents allow chemical absorption to take place [31,32]. Examples of some of these solvents used for chemical absorption are amines, chilled methanol and ammonia solutions [33,34,35]. Whilst absorption may be considered a mature technique, it has its drawbacks, such as (i) the loading capacity of CO_2_ being dependent and limited by the solvent; (ii) the solvents promoting corrosion in the equipment; (iii) the regeneration of solvent requiring high energy, leading to this not being economically viable; (iv) evaporation causing a significant loss of solvent and (v) the oxygen-rich environment degrading the solvent [27].

### 3.2. Adsorption

This is a selective process that allows the molecules in the gaseous mixture to adhere to a solid surface known as an adsorbent. The quality of the adsorption is determined by the properties of the adsorbed particles and the adsorbent [27]. Regardless of the CO_2_ particle size during the CO_2_ capture process, the particles can be adsorbed using the appropriate adsorbents, such as zeolites, metal oxides, porous silicates and metal organic frameworks [36]. The process takes two stages into account, which are adsorption and desorption. However, the adsorption process is exothermic, meaning that regeneration of the adsorbents through desorption can be carried out by increasing the temperature [27]. Further, adsorption captures particles easily, but this could consequently result in harder desorption, as more energy is needed to release the particles captured. In order to ensure the efficiency of adsorption and desorption, many power plants use activated carbon fibres and carbon fibre components [37].

Additionally, the adsorption technology is attractive to use due to its low energy requirements, flexibility, simplicity to operate and ease of maintenance [38]. On the other hand, adsorption technology in the pack bed and slow kinetics has poor heat, which makes it disadvantageous [18].

### 3.3. Cryogenic Distillation

This process requires a stream with a high concentration of CO_2_, as it applies the concepts of condensation and cooling [39,40]. Cryogenic distillation occurs at low temperatures and allows the gas mixture to separate via fractional condensation [41,42]. This technique does not require any solvents or sorbents, which makes it advantageous, as no costs are incurred [27,43]. However, it needs to functionalised at low temperatures, making it energy-intensive, which, in turn, increases the operating costs [18]. Furthermore, as flue gas has low concentrations of CO_2_, it is not be ideal to use this process, as the low temperatures will compromise equipment safety [44,45]. As such, an economically viable, less energy-intensive separation process technique needs to be explored, such as membrane separation [46].

### 3.4. Membrane Separation

This pressure-driven technique allows the difference in partial pressure between the feed and the permeate sides to create a driving force for separation to occur [47]. Membrane technology has no phase change, which leads to efficient volume and weight when applied to industrial units. Further, chemicals are not used, which means no operating costs, thereby allowing scalability. Moreover, it has lower capital costs compared with the other three process techniques [5]. While these may be advantageous, the performance of the membrane for CO_2_ separation is dependent on the type of materials utilised in fabrication, and CO_2_ permeability (*P_A_*) and selectivity (α_AB_) [48]. Other factors include the membrane’s structure and thickness, which determine the permeance; the system design and the configuration of the membrane. Furthermore, other than the membrane, the feed concentration of CO_2_ is crucial, as the partial pressure can be affected [49]. Hence, finding the ideal configuration of selectivity and permeability is vital in a membrane set-up. As a result, many researchers are debating whether to use high permeance and low selectivity or low permeance and high selectivity [50]. Overall, the efficiency of CO_2_ capture with membrane technology depends on the following criteria: high CO_2_/N_2_ selectivity, high CO_2_ permeance, chemical and thermal robustness, good resistance to aging and plasticisation, and cost-effective with low manufacturing costs [51].

To conclude, the advantages and disadvantages of these techniques have been summarized in Table 1.

## 4. Membrane Gas Separating Technology

**Figure 1:**
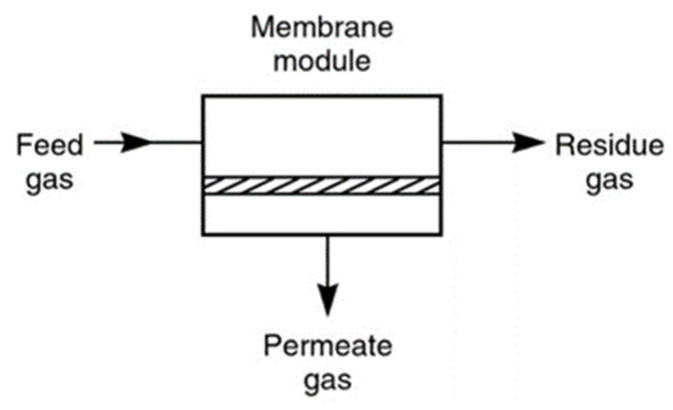
Schematic of membrane gas separation process. The figure reproduced with permission from [62] Access Date: 14 August 2020.

Over the years, chemical separation using membrane technology has attracted enormous interest in fields such as gas separation, water purification, food processing, pervaporation, membrane contactors and reactors, and so on [53,54]. Membrane technology has lower overall and operational costs, thereby allowing it to compete with processes such as absorption, adsorption and cryogenic distillation [55,56]. In terms of economic viability in CO_2_ separation, membrane technology has lower overall costs [57]. The 1980s showed the first successful industrial application of membrane technology, which was used to separate hydrogen from nitrogen, argon (Ar) and methane (CH_4_) in an ammonia (NH_3_) plant [58,59]. Furthermore, membrane technology is able to generate high selectivity of CO_2_ from the flue gas without needing a high concentration of CO_2_ in the inlet [27]. Additionally, membrane separation uses smaller operation units, lacks mechanical complexity and eases scaling up and installation [36,60,61]. Looking from a monetary standpoint, it also lowers capital and operating costs, and introduces an environmentally friendly element as well [62,63]. Figure 1 displays pressure-driven membrane separation using permeable or semi-permeable phases. By using such phases, membrane separation allows certain components to pass through or restrict the movement of other undesirable components. Ideally, the membrane can be seen as a barrier between the feed stream and the product stream [64,65].

**Figure 2:**
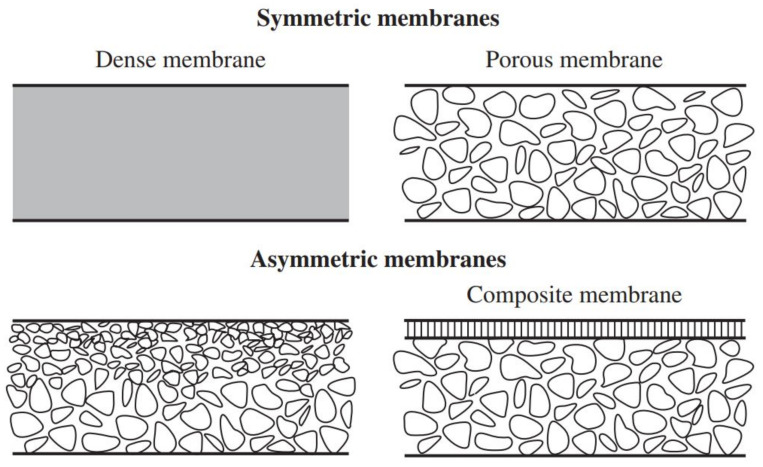
Schematic of two membrane structures. The figure reproduced with permission from [62] Access Date: 14 August 2020.

In membrane separation, the internal structure or morphology is a significant criterion of how the membrane can be used and how effective it is. As shown in Figure 2, there are two categories of internal structure for membranes: isotropic (symmetric) and anisotropic (asymmetric) [66].

The first type (isotropic) is a dense microporous (non-porous) membrane, which has a low flux due to its thickness and small pore size. This type of membrane is used in labs rather than in industrial processes [67,68]. The second type (anisotropic) has a higher flux due to its bigger pore size, which is not uniform and differs according to the location. As such, composite membranes have a thin top layer, resulting in higher permeance and selectivity. Hence, due to these qualities, anisotropic membranes are preferred and practical to use in various industries [66]. Table 2 summarises the pore diameters of membranes that are reported in the literature.

## 5. Type of Membranes

The membrane-based separation method has slowly gained recognition, as it is a promising option because it is economical, efficient and environmental friendly for CO_2_ capture and separation [70]. There are three types of membranes, namely polymeric membranes, inorganic membranes and mixed matrix membranes (MMMs). Each of these membranes are further categorised into porous and non-porous membranes [53].

### 5.1. Polymeric Membranes

There are two different polymeric membranes, which are glassy and rubbery. These membranes are also known to exhibit an inverse proportionality of permeability and selectivity. This means that when selectivity increases, the gas permeability tends to decrease [71,72]. This is because polymeric membranes are very thin, which allows them to achieve high gas permeability [47]. Rubbery membranes are soft, flexible and elastic, and have the ability to operate above their own glass transition temperatures [49]. Additionally, rubbery membranes display low selectivity but higher permeability [71]. Due to this reason, rubbery membranes are rarely used in industries because of their lack of selective properties. Therefore, glass membranes are preferred for the following reasons [43]. Glassy membranes are hard and rigid, and are able to operate below their own glass transition temperatures [73]. In addition, glass membranes have selective and permeability properties opposite to those of rubbery membranes. Glass membranes have higher selectivity and low gas permeability [27,71].

### 5.2. Inorganics

In the fabrication process of inorganic membranes, materials such as carbon, metals and ceramics are embedded [74]. Ceramics and metals are included in these types of membranes to increase the mechanical strength with minimum resistance in mass transfer. These membranes used in CO_2_ separation possess properties that increase their thermal and chemical stability and reduce their selectivity and permeability when compared with other membranes [11,75]. Further, inorganic membranes are expensive and complicated to fabricate. Moreover, these membranes are brittle and fragile, making them harder to handle, and thus are not ideal for the current situation [76]. A typical example of an inorganic membrane is the silica membrane, which can selectively isolate CO_2_ from flue gas. In addition, zeolite membranes may be used for CO_2_ capture, but this approach is not as advanced as the polymeric CO_2_ capture techniques [77]. The efficiency of an inorganic membrane is characterised by its properties, such as porous structure, pore width, pore texture, surface roughness, hydrophobicity and tortuosity [47].

### 5.3. Mixed Matrix Membranes (MMMs)

It can be concluded that between polymeric and inorganic membranes, polymeric membranes have higher economic benefits due to their flexible physical structure and solution processability [78]. However, a critical disadvantage of polymeric membranes is the trade-off trend restriction between selectivity and permeability. Polymeric membranes are inadequate in applications that are of an industrial scale [79]. Therefore, an alternative form of enhanced gas separation performance would be the utilisation of MMMs, which are above Robeson’s upper bound line [80]. This membrane is fabricated with a filler that is distributed uniformly in a polymer matrix [81]. The properties of the fillers and polymer materials directly affect the morphology and separation performance of MMMs [70]. Paul and Kemp first discovered the concept of MMMs in the early 1970s. They observed that a significant increase in the diffusion time lag of CO_2_ increased the separation performance when 5A zeolites were added to a rubbery polymer membrane known as polydimethylsiloxane (PDMS). In light of these results, further research focused on composite membrane structures with the incorporation of various fillers to aid in the separation performance of MMMs [80,82]

**Figure 3:**
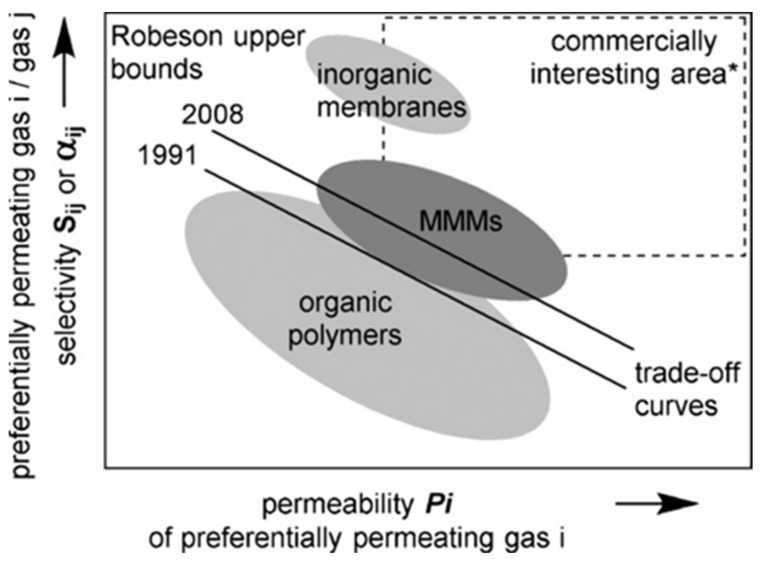
Selectivity of all three types of membranes from the Robeson plot between permeability and selectivity [83].

MMMs are an up-and-coming category of membranes, which allow higher selectivity and permeability, and are much more advantageous than polymeric membranes. Figure 3 illustrates the relationship between selectivity and permeability in the three types of membranes in Robeson’s plot [83]. As inorganic membranes are expensive to fabricate in industrial applications, MMMs are utilised due to their advantages [84].

The MMMs are fabricated from organic polymer layers and inorganic filler particles. These two heterogeneous membranes can only coexist through synergistic interactions between them [85]. The inorganic layer acts as the dispersed phase and the polymer layer as the bulk phase. Through further research by Rezakazemi and coworkers, it was found that the conventional inorganic fillers used are zeolite particles, carbon nanotubes (CNTs) and silica-type particles [86].

MMMs allow the trade-off between selectivity and permeability in pure polymer membranes to be broken [70]. This is because MMMs balance the benefits of the mechanical properties, processability and cost of polymers with the power of fillers in terms of permeability and selectivity. This is essentially based on the form of the material selected for the MMM. The polymer–inorganic composite membrane system allows much more effective isolation of gas, and prevents blockage of the pores, accumulation of voids, agglomeration of particles and regasification of polymers [70].

In summary, the advantages and disadvantages of these membranes have been tabulated in Table 3, as shown below:

## 6. Limitations of MMMs

**Figure 4:**
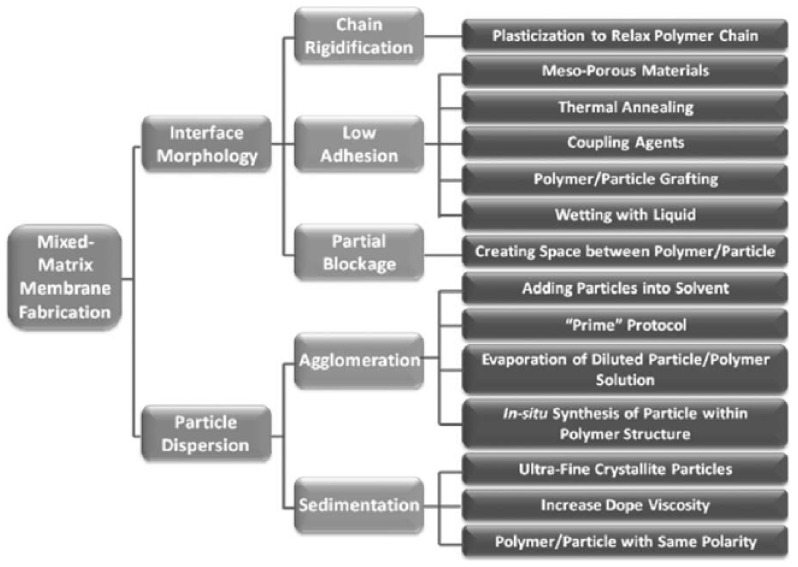
Overview of the challenges in MMMs [87].

There are several challenges faced during the fabrication of MMMs, such as the desired morphology, gas separation properties and mechanical/chemical stability [87]. Some of these challenges are: (i) voiding the loss of selectivity as a result of agglomeration by achieving a homogeneous dispersion of particles, (ii) guaranteeing the membrane’s integrity and separation performance by ensuring a defect-free polymer/inorganic particle interface, and (iii) selecting the polymer and inorganic fillers based on compatibilities and keeping a strong separation property in mind. Figure 4 shows the challenges and solutions faced in the fabrication of MMMs [87]. Additionally, in the fabrication of MMMs, homogeneous particle dispersion is crucial during synthesis because obtaining a balance between the polymer matrix and inorganic fillers influences the effectiveness of the membrane [87]. However, when inorganic fillers are used, loading is a concern, as there is a formation of accumulated particles. When this occurs, a void is created where it does not connect to the polymer chain. As a result, selectivity is low and gas is allowed to move through these voids [88]. As such, a blend with solvents can help with this challenge [64]. Finally, appropriate selection of membrane materials plays a vital role in overcoming these challenges. The selection should be dependent on the final purpose, which is synthesising a MMM with high selectivity and permeance during the performance of CO_2_/N_2_ separation [87].

Choosing an ideal membrane would require: (i) high selectivity and (ii) high permeability of CO_2_ molecules. However, in many common cases, this is not always true. It has been recognised that both these conditions—permeability and selectivity—are inversely related [89]. The upper bound is the trade-off between the correlation of pure gas selectivity and lighter/faster gas selectivity [79]. In polymeric membranes, this trade-off is often noticeable, where an inverse relationship between selectivity and permeability is experienced. Thus, the selectivity of the membrane of different gas pairs increases and the permeability decreases [90]. The advantage of using MMMs is that they have the ability to overcome the upper bound limit, hence allowing the membrane to have high permeability along with high selectivity [80].

## 7. Blended MMMs

The MMMs are considered as upcoming membranes for gas purification [91]. There has been interest in blended MMMs because of their unique ability and the characteristic of using polymers and inorganic fillers [91,92]. Further research has shown that there has been evidence of their potential to perform better without having to increase the cost of fabrication [53]. Moreover, fabricating blended MMMs along with an appropriate loading of inorganic fillers and polymer blends allows synthetisation, resulting in high permeability and selectivity of CO_2_ [93,94]. The loading percentage of the fillers and the properties of the polymer matrix and solvents are the main factors that affect the fabrication and ability of the blended MMMs [95]. A drawback of using blended MMMs is finding an inorganic filler that is compatible with different polymers and is close to the CO_2_/N_2_ upper bound limits [81]. Thus, affecting the morphology of the membrane consequently affects the membrane’s effectiveness [96].

## 8. Membrane Materials

### 8.1. Polymers

Both polyether sulfone (PES) and polyethylene glycol (PEG) are different types of polymer material. Each of these polymers has its own set of characteristics, making them unique in the process of CO_2_/N_2_ separation [97]. PES is a glassy polymer and PEG is a rubbery polymer [64,98]. Each of these polymers is applied to different applications [95]. PES is commonly applied for thermal stability, gas separation properties and processability due to its glassy nature. On the other hand, PEG allows permeability for CO_2_ and selectivity for N_2_ because of its rubbery material [99]. Since 2016, the removal of CO_2_ from natural gas has been the only membrane-based separation being carried out on a large scale [100]. PEG acts as a driving force, pushing the gas through the membrane due to the difference in concentration or pressure. Hence, as PES has high gas separation properties and PEG has high CO_2_ and N_2_ permeability, both polymers are used for CO_2_/N_2_ separation [101].

### 8.2. Solvents

The morphology of membranes is strongly affected by the solvents used during fabrication [102]. Membrane morphology performance is important, as it directly affects the manner in which the filtration application occurs [43,103]. Additionally, different types of solvent can cause a change in morphology, such as pore size, and allow different rejection rates to be experienced by the membrane [103,104]. NMP solvents are used, as these have been shown to improve the permeability of CO_2_ as well as reduce the non-selective voids and increase the gas selectivity [105]. This solvent also assists with the dispersion of MWCNTs-F, as it has similar solubility parameters to CNTs. In addition, NMP allows the formation of a membrane when two polymers are utilised [106,107]. Further, another solvent known as dimethylformamide (DMF) has solubility parameters similar to those of polymers. This solvent rearranges polymeric chains and produces a lower thermodynamically entropy, and forms a structure low in permeability and high in selectivity of CO_2_ and N_2_ [102,105].

In a comparative study, Ahmad and coworkers studied the gas permeation and morphology of dense PES membranes using three solvents, namely DMAc, DMF and NMP. Their study concluded that the morphology of the three membranes produced dense structures and were able to diffuse the permeation through pressure, concentration or the potential gradient [105]. This showed that the PES–DMF membrane obtained a CO_2_/CH_4_ selectivity of 2.56 compared with the PES-DMAc (2.13) and PES-NMP (2.4) membranes [105]. The DMF solvent has a low density and viscosity compared with water; thus, it is more efficient and is capable of CO_2_ solubility [108]. Hence, both NMP and DMF are used to increase the selectivity of CO_2_/N_2_ separation due to the different phase inversion processes [105,108,109].

### 8.3. Fillers

The morphology and performance of membranes are significantly affected by parameters such as the type of polymer matrix and the inorganic fillers interacting between the two phases [110]. By using the appropriate inorganic filler material, the MMM’s permeability as a whole is enhanced and the transport properties of gases is improved [111]. With the presence of fillers, the fabrication costs are lowered [95]. On the other hand, not every combination gives a positive result. Some combinations may cause the MMM to act poorly and even reverse the effects [6].

Non-porous organic fillers increase the matrix pattern and decrease the diffusion layer, thus increasing the separation performance of the MMM [112]. A common addition to MMMs is silica in a polymer matrix, which can alter the polymer chain, resulting in an increase in O_2_ and N_2_ permeation. However, due to surface chemistry and the distribution of pore sizes, porous inorganic filler materials are more compatible with a polymer matrix. This results in higher efficiency in gas separation compared with using non-porous inorganic fillers [6]. In terms of the surface chemistry of a non-porous organic filler, not only is the matrix’s tortuous pattern enhanced but a molecular sieve is created, which then separates gas particles based on size and shape [113]. This, in turn, creates high gas permeability or the desired component selectivity [113]. Therefore, the introduction of porous inorganic fillers to the polymer matrix not only enhances the permeability of the target species but also increases selectivity [114].

Other commonly used inorganic fillers include metal oxide, silica and carbon molecular sieves (CMS) [86]. However, as mentioned previously, some combinations with the polymer matrix can give poor results by lowering the selectivity and permeance. Furthermore, these inorganic fillers may not improve the separation performance sufficiently [110]. As such, researchers are designing MMMs which can disperse a combination of nano-sized fillers, resulting in better contact with the polymer matrix [6]. Hence, a more robust CO_2_ separation is created. A prime example of these are carbon nanotubes (CNTs) [115].

### 8.4. Functionalized Carbon Nanotubes (CNTs)

**Figure 5:**
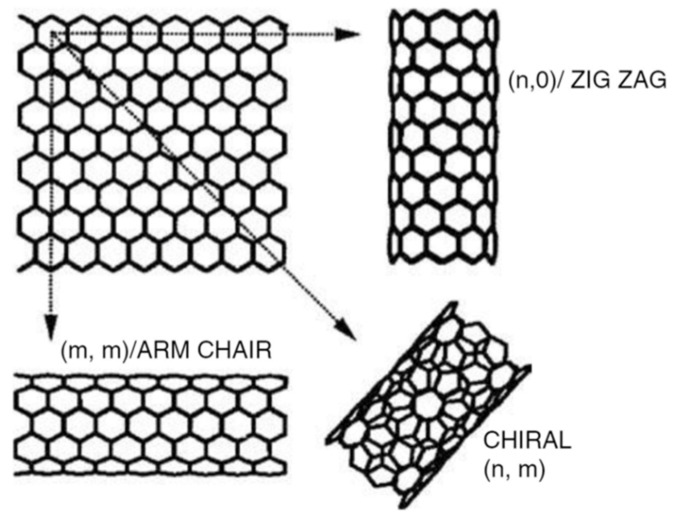
Various structures of CNTs [69].

Carbon nanotubes (CNTs) in MMMs show a robust and promising possibility of overcoming the trade-off of selectivity and permeability, which is the primary issue experienced in MMMs [113,114,115,116]. CNTs also have a higher rapid gas transport rate that allows for higher permeability compared with the other fillers mentioned [117,118]. Additionally, the polymer matrix has a stronger interaction with functionalised CNTs in the MMMs [119]. Besides, CNTs have more durable mechanical properties due to the carbon–carbon bond found in the graphite layers [115], as demonstrated in Figure 5. Furthermore, together with this graphite layer, CNTs have also a secure honeycomb cylindrical lattice structure, resulting in strong mechanical strength even at low concentrations [6].

To summarized, the up to date blend membrane works have been tabulated in Table 4. 

## 9. Fabrication Method

The fabrication method of membrane is dependent on the type of desired membrane (isotropic or anisotropic) and the materials used for synthesizing [122]. Once the materials have been chosen, there are three conventional methods of membrane fabrication: stretching, track-etching and phase inversion.

### 9.1. Stretching

The first fabrication method is called stretching, which is a technique that is applied to semi-crystalline polymer materials. The semi-crystalline polymer materials go through the process of extruding and stretching [122]. The process is a solvent-free technique where the polymer gets heated past its melting point and extruded until a thin sheet is formed, followed by stretching [123,124,125,126]. Stretching has two steps, the first being cold stretching, which allows the creation of micropores in the thin film. The pores in the membrane’s final structure is then controlled by hot stretching, which is the second step [122]. In this process, the materials’ physical properties and the processing parameters control the final porous structure and properties of the membranes [122,127].

### 9.2. Track Etching

**Figure 6:**
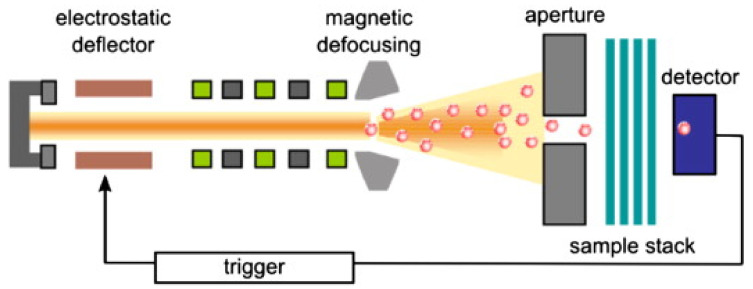
Schematic of fabrication via track etching using a single ion irradiation set-up [122].

The next technique is track etching, where a non-porous polymer film is irradiated with energetic heavy ions, leading to linear impaired tracks in the irradiated polymer film [128]. This fabrication technique is known for its precise control of the membrane’s pore size distribution; pore size and pore density are independent parameters and can be controlled from a few nanometres to tens of micrometres and 1–10^10^ cm^−2^, respectively [122]. Chemical etching is accompanied by a uniform quasi-cylindrical pore diameter. The diameter can be modified by adjusting the etching time. Furthermore, the number of pores can be calculated using irradiation ion fluence [129]. An example of track etching where a single ion irradiation system is used to produce a track-engraved membrane, is provided in Figure 6 [122].

### 9.3. Phase Inversion

**Figure 7:**
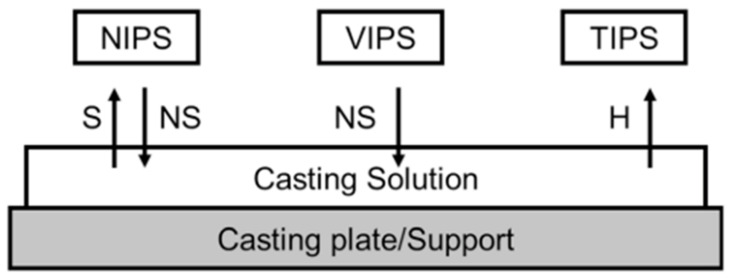
Schematic of phase inversion [69].

Phase inversion is a demixing process, which transforms liquids to solids in a controlled manner from a homogeneous polymer solution [122]. The polymer solution is submerged in a non-solvent (typically water) coagulation tank. Demixing and precipitation occur due to an interaction between the solvent (from a polymer solution) and the non-solvent (from a coagulation bath), i.e., the solvent and non-solvent must be miscible [123]. Thermally induced phase separation (TIPS) is an approach the focuses on the phenomenon that generally decreases solvent content when the temperature is lowered. Once demixing has been triggered, precipitation, evaporation or freeze-drying eliminates the water [124]. During evaporation-induced phase separation (EIPS), the polymer solution is produced from a solvent or a combination of a volatile non-solvent and the solvent, which can evaporate, contributing to precipitation or demixing/precipitation. This procedure is also regarded as a method for casting solutions [125]. In vapour-induced phase separation (VIPS), the polymer solution is subjected to a non-solvent atmosphere, and the accumulation of the non-solvent induces demixing/precipitation [122]. Figure 7 illustrated the fabrication method of this process. 

## 10. Transport Mechanism

In membrane technology, a common gas separation transport mechanism used is diffusion. This is the movement of gaseous components from a higher concentration to a lower concentration until equilibrium is reached [43,130]. However, depending on the membranes used, there are different types of mechanisms that can be utilised: the Hagen–Poiseuille mechanism, Knudsen diffusion and molecular sieving [69]. The performance of many of these mechanisms is heavily dependent on the gases involved, the membrane properties, the operating pressure and the operating temperature [131].

### 10.1. Hagen–Poiseuille Mechanism

The Hagen–Poiseuille mechanism is used if the pore sizes are from 200 nm to 3000 nm and bigger compared with the transporting molecules’ free path. This mechanism is driven by the pressure gradient between the two sides [132]. Additionally, this mechanism applies the idea of average velocity to induce more gas molecules to collide with each other, resulting in more transportation through the pores. Thus, the mechanism is independent of the shape, mass and size of the molecules [133,134]. As such, the Hagen–Poiseuille mechanism is ideal for the transportation of bulk flow of fluids [69].

### 10.2. Knudsen Diffusion

Knudsen diffusion occurs when the pore sizes are smaller than the free path of the transporting molecules [69,132,135]. This is ideal for diffusing gas molecules, as it creates more collisions against the pore walls rather than the molecules itself, where the small diameter of large pores regularly collide with the wall [115]. Additionally, this type of diffusion occurs when the pore sizes (*r*) are smaller than the gas molecules in the free path (*λ*), which is shown in Equation (1) [136].

(1) $$\lambda =\frac{3\eta }{2p}\frac{{\left(\pi RT\right)}^{\frac{1}{2}}}{2M}$$

In Equation (3), η is the gas velocity, *R* is the universal gas constant, *T* is the absolute temperature, *P* is the pressure and *M* is the molar mass. This equation further illustrates that when the membrane pore size is smaller than *λ* (*r*/*λ*, 0.05), more wall collisions occur compared with collisions among the molecules. This means that the molecules are moving independently. Based on this concept, gas separation is carried out through the velocity differences of the different component molecules, where the lighter ones go through the membrane. As such, the molar flux is calculated as shown in Equation (2) [136], where G_Knudsen_ is the molecular flow of the gas, r is the pore radius, *P*_1_ is the partial pressure on the feed side gas, *P*_2_ is the partial pressure of the permeate side gas, *L* is the pore length, *M* is the molar mass, *R* is a gas constant and *T* is the absolute pressure.

(2) $$G_{Knudsen}=\frac{8r\left(p_{1}-p_{2}\right)}{3L{\left(2\pi MRT\right)}^{\frac{1}{2}}}$$

Furthermore, the Knudsen mechanism’s selectivity of separation is predicted from the ratio of the molecular weights and its square root, which is shown in Equation (3) [136].

(3) $$\alpha K_{n}=\sqrt{\frac{M_{j}}{M_{i}}}$$

### 10.3. Molecular Sieving

The last method of transportation is molecular sieving, where the sizes of both the transported molecules and pores are very similar. This means that molecules of a larger size would not be able to transport through the membrane [137].

## 11. Conclusions

As PEG has a polar ether group presence, it can be concluded that this polymer has a strong affinity towards CO_2,_ which, in turn, increases the selectivity of CO_2_. In addition, together with PES, the mechanical and chemical stability of the membrane is improved. Further, PES is in the ether-oxygen group, which binds CO_2_, thus increasing the selectivity again. On the other hand, blended NMP and DMF reduce non-selective void formation in the membrane and cause low density, respectively, which cause an improvement in CO_2_ permeability and solubility. Finally, by utilising MWCNTs-F, the permeability and selectivity of the membrane can be enhanced, thereby allowing faster gas transportation across the membrane. Hence, a blended MMM fabricated using PES, PEG, NMP, DMP and MWCNTs-F is believed to have a desirable effect on CO_2_/N_2_ separation. Lastly, the challenges of developing this new blended MMM such as cost and compatibilities should be further considered.

## 12. Future Prospects

The following conceptual prospect shows that blending different polymers and solvents to fabricate MMMs would overcome the trade-off limitations between selectivity and permeability. This is due to the combination of the benefits of polymeric and inorganic membranes, and the morphology created by the solvents. Moreover, with the introduction of inorganic fillers, the gas separation properties can be improved. However, an abundance of different combinations of possible polymers, solvents and fillers remains to be discovered and researched. Thus, future research should be focused on studying different combinations of polymers, solvents and inorganic fillers by utilising the concept of blending and MMM fabrication to synthesise a membrane capable of breaking the trade-off limitations. Additionally, a future prospect for a proposed blended MMM concept would be to conduct a gas separation study along with a kinetic study to better understand the effects of the chosen polymers and inorganic fillers, and the resulting selectivity and permeation. These outcomes will expand growth in the membrane technology field and allow researchers to further advance in this field.

## Figures and Tables

**Table 1:** Advantages and disadvantages of CO_2_ separation techniques [52].

Processes	Advantages	Disadvantages
Absorption	High efficiencies of absorption (>90%)	Efficiency of absorption is highly dependent on CO_2_ concentrations
	Sorbents can be regenerated through depressurisation and heating	A large amount of heat is essential for the regeneration of sorbents
	Most advanced technology for CO_2_ separation	Must fully understand the impacts of degradation of the sorbents on the environment
Adsorption	Reversible process and recyclable absorbents	High-temperature adsorbents are needed
	High efficiencies of adsorption (>85%)	Require high energy for desorption of CO_2_
Cryogenic Distillation	Technology implemented for many years for CO_2_ recovery	Feasible only for a high concentration of CO_2_ (>90% *v*/*v*)
		Must be applied at extremely low temperatures
		Highly energy-intensive technology
Membrane Separations	High efficiencies of separation (>80%)	Permeability and selectivity balance

**Table 2:** Pore diameter categories.

Category	Pore Diameter	References
Macroporous	>50 nm	[67]
Mesoporous	2–50 nm	[66]
Microporous	1–2 nm	[69]
Nanoporous	<1 nm	[68]

**Table 3:** Advantages and disadvantages of different membranes in gas separation [6].

Membranes	Advantages	Disadvantages
Polymeric Membranes	Easy synthesis and fabrication	Low thermal and chemical stability
	Low production cost	Plasticisation
	Good mechanical stability	Pore size cannot be adjusted
	Easy to scale up	Follows the trade-off between selectivity and permeability
Inorganic Membranes	Stronger chemical, mechanical and thermal stability	Brittle
	Pore size is adjustable	Expensive
	Able to work in harsh conditions	Difficult to scale up
	Moderate trade-off between selectivity and permeability	
Mixed Matrix Membranes	Better mechanical and thermal stability	The high fraction of fillers renders it fragile in the polymer matrix
	Lower plasticisation	The quality of the polymeric matrix dictates the chemical and thermal stability
	Lower energy requirement	
	Compacts at a higher pressure	
	Exceeds the trade-off between selectivity and permeability	
	Separation is accomplished by the concept of hybrid polymeric and inorganic membranes	
	Superior separation performance over the typically used polymeric membranes	

**Table 4:** Reproduced summary of reported blended membranes [69].

Polymer Pair	Application A/B	A Permeability, P_A_	A/B Selectivity, α_A/B_	Researcher
PEG-400/PTFPMS	CO_2_/N_2_	56.27 ^α^	26.67	Nie et al. (2013) [62]
PSF/PEI	CO_2_/CH_4_	~4.59 ^β^	~11.45	Mukhtar et al. (2016) [120]
PES/PVAc	CO_2_/CH_4_	120.23 ^α^	16.96	Farnam et al. (2016) [100]
PES/PEG—10,000	CO_2_/N_2_	~5.26 ^β^	~40.79	Akbarian et al. (2018) [64]
PU/PVA-200	CO_2_/N_2_	93.24 ^β^	32.6	Shirvani et al. (2018) [121]
	CO_2_/CH_4_	93.24 ^β^	9.49	

^α^ GPU, ^β^ Barrer.

## Data Availability

Not applicable.
